# Modulation of Steroid and Triterpenoid Metabolism in *Calendula officinalis* Plants and Hairy Root Cultures Exposed to Cadmium Stress

**DOI:** 10.3390/ijms23105640

**Published:** 2022-05-18

**Authors:** Agata Rogowska, Cezary Pączkowski, Anna Szakiel

**Affiliations:** Department of Plant Biochemistry, Faculty of Biology, University of Warsaw, 1 Miecznikowa Street, 02-096 Warsaw, Poland; myhacp@biol.uw.edu.pl (C.P.); a.szakiel@uw.edu.pl (A.S.)

**Keywords:** abiotic stress, *Calendula officinalis*, hairy roots, sterols, triterpenoids

## Abstract

The present study investigated the changes in the content of steroids and triterpenoids in *C. officinalis* hairy root cultures and plants exposed to cadmium stress. The observed effects included the content and composition of analyzed groups of compounds, particularly the proportions among individual sterols (e.g., stigmasterol-to-sitosterol ratio), their ester and glycoside conjugates. The total sterol content increased in roots (by 30%) and hairy root culture (by 44%), whereas it decreased in shoots (by 15%); moreover, these effects were inversely correlated with Cd-induced growth suppression. Metabolic alterations of sterols and their forms seemed to play a greater role in the response to Cd stress in roots than in shoots. The symptoms of the competition between general metabolites (sterols) and specialized metabolites (triterpenoids) were also observed, i.e., the increase of the sterol biosynthesis parallel to the decrease of the triterpenoid content in *C. officinalis* plant roots and hairy root culture, and the inverse phenomenon in shoots. The similarity of the metabolic modifications observed in the present study on *C. officinalis* plant roots and hairy roots confirmed the possibility of application of plant in vitro cultures in initial studies for physiological research on plant response to environmental stresses.

## 1. Introduction

Metal ions are inherent elements that enable the course of physiological processes in living organisms. Besides macronutrients such as Ca^2+^, Mg^2+^, or K^+^ ions, plants also require micronutrients for proper development, including heavy metals. Some heavy metal ions play important roles in biochemical processes in plant cells, such as Cu^2+^ ions, which are involved in electron transport reactions in photosynthesis and respiration [1]. However, most heavy metals are nonessential for plants and may negatively impact their growth and development, even in small quantities. Among the various heavy metals, Cd is considered the most phytotoxic one. Cadmium salts are soluble in water, and Cd ions are easily absorbed by plants, thus entering the food chain [2,3]. Cadmium toxicity is represented by the replacement of biogenic metals in the active centers of enzymes and the high affinity to the thiol groups of proteins, which inhibits enzyme activity [4,5]. Cadmium toxicity in plants is manifested by stunted growth and chlorosis; at higher concentrations, it can lead to growth inhibition and necrosis [6,7] as well as reduced photosynthetic activity by decreasing the chlorophyll content [8]. As soil is the main sink for heavy metals, plant roots are the most important organs in metal ion uptake and relocation. Roots are also the first organs directly affected by cadmium damage, disturbing the normal physiological metabolism of the entire plant [7,9].

A critical step in plant survival strategy during exposure to external stress is maintaining the fluidity and integrity of the cell membranes [10]. As sterols are plant membrane components, changes in the profiles of these compounds seems to be important as a response to adverse environmental conditions. Sterols not only perform a structural function along with sphingolipids and glycerolipids, but also influence the activity of integral membrane proteins, including enzymes, ion channels, receptors, and components of signal transduction pathways (i.e., ATPases). These isoprenoids are regarded as primary or, more aptly, general metabolites due to their metabolic, regulatory, and membrane architectural functions [11]. Cell membranes are important sites perceiving abiotic stress signals, and, therefore, the recomposition of endogenous sterols in these structures improves plant stress resistance. A specific role in this process is played by C24-alkyl sterols, which enhance plant resistance to stress factors by improving the cohesion of cell membranes and changing the permeability of membranes, thereby affecting proton efflux [12,13]. The proportions between sitosterol and stigmasterol (which contains 22(23)-double bond in the side chain) and the ratios of certain conjugated sterol forms (i.e., sterol esters and sterol glycosides) can change under abiotic stress [11]. In sterol esters forms, fatty acids estrified to C3 hydroxyl group may vary in lengths from twelve carbon atoms to twenty-two. The most common fatty acids found in sterol esters are palmitic, stearic, oleic, linoleic, and linolenic. In the case of glycosylated sterols, sugar moiety is linked through β-glycosidic bond to C3 hydroxyl group of sterol core. The most common monosaccharide found in plant glycosylated sterols is glucose; however, other monosaccharides, such as galactose, xylose, and mannose, have also been identified [14].

Another interesting aspect of plant response to stress is the relationship between general and secondary metabolism. Specialized metabolites deal with various external agents (including stress factors), protection against herbivores, or attraction of pollinators. Some stress conditions enhance the expression of genes involved in the biosynthesis of certain natural defense compounds [15]. Sterols, considered general metabolites, and triterpenoids, which are specialized compounds, have a common precursor, namely, squalene. Therefore, the metabolic strategy of the plant facing stress factors can be regarded as a kind of compromise: either the boost of the biosynthesis of specialized defense metabolites at the expense of general metabolites, which may result in growth inhibition, or enhanced biosynthesis of general metabolites to better adapt to adverse conditions, along with the reduced production of specialized metabolites.

Cadmium uptake by roots has been extensively studied in various important crop species, including cereals (maize, rice, and wheat) and vegetables (lettuce, radish, and onion). These studies provide evidence that cadmium ions directly affect the physical properties and biological functions of cell membranes in roots, along with an indirect effect via reactive oxygen species formation [16]. However, little is known about the effect of cadmium stress on cell membrane composition in the context of possible alterations in sterol content. To date, only limited data are available on the influence of cadmium ions on sterols and secondary metabolites in *Calendula officinalis* L. (calendula, marigold), a plant with ornamental and medicinal value and native to the south of Europe. Due to its high adaptability, it does not require specific soil conditions and seems to be a promising candidate for cadmium phytoremediation [17,18,19].

The present study investigated the changes in the contents of steroids and triterpenoids in *C. officinalis* hairy root cultures and plants exposed to cadmium stress over time. Apart from the evaluation of the influence of cadmium ions on steroid and triterpenoid biosynthesis (and, thus, the potential trade-off between general and specialized metabolism), the additional aim of this study was to compare the observed effects in two different experimental models. Investigations of in vitro cultures are convenient due to the controlled environmental conditions as well as the less complex culture maintenance and supplementation with stress agents or elicitors. However, the common critical reproach against such studies is that in vitro cultures might not reflect the stress response of the plant under natural conditions. In this context, this study complements the knowledge on the usability of plant in vitro cultures as experimental models for research on physiological stress reactions, and not only for typical studies on plant productivity of valuable bioactive phytochemicals.

## 2. Results

### 2.1. Cadmium Influence on C. officinalis Hairy Root Culture

A *C. officinalis* hairy root culture was supplemented with cadmium ions as described in Section 4.2.1. The concentration of cadmium ions (25 µmol/L) was chosen based on previous findings [20] to allow the observation of the exerted effects, simultaneously avoiding the strongest toxicity that might lead to the decay of the root culture. Growth parameters, cadmium accumulation in hairy root tissue, as well as the contents of steroids and triterpenoids were determined at 3, 7, 14, and 21 days after treatment to evaluate the course of the stress response over time.

#### 2.1.1. Cadmium Effect on Hairy Root Biomass

**Figure 1:**
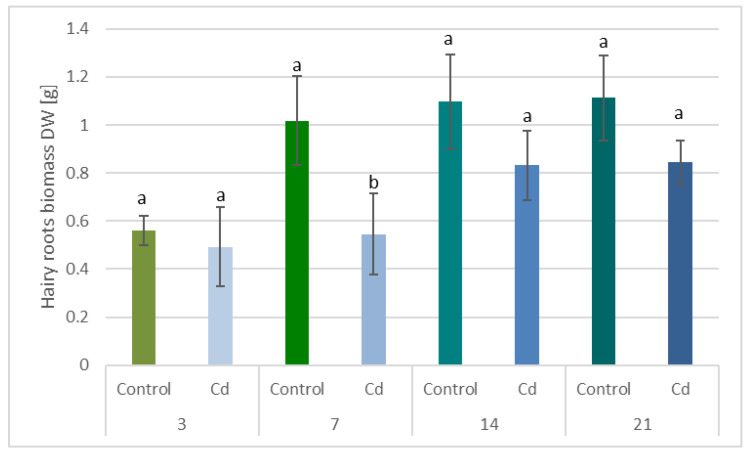
The time-dependent effect of cadmium ions on hairy roots biomass. Bars which do not share a common letter are significantly different (*p* < 0.05).

**Figure 2:**
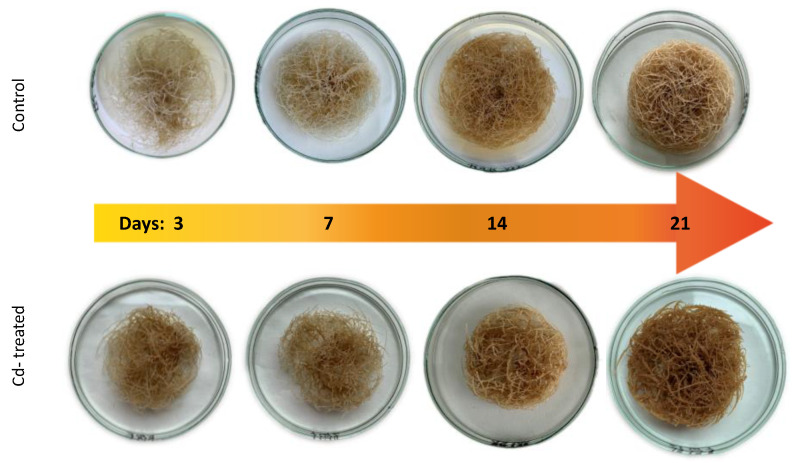
Cadmium-induced morphological changes in hairy roots.

The presence of cadmium in the growth medium significantly influenced the growth of *C. officinalis* hairy roots, i.e., in Cd-treated samples, reduced biomass growth, alterations in branching, and browning of tissues were observed. The time-dependent effect of cadmium ions on hairy root biomass (expressed as dry weight, DW) is shown in Figure 1 and Figure 2. Growth inhibition was only slight (12%) at the beginning of the experiment (3 days after treatment), whereas the strongest effect, i.e., a decrease in hairy root biomass by 46% in Cd-treated samples, was noticed after 7 days of incubation with Cd ions. With an increased incubation period, the difference between the control and Cd-treated samples diminished to 24% (in comparison with the respective control samples), both after 14 and 21 days.

#### 2.1.2. Accumulation of Cadmium in Hairy Roots

**Figure 3:**
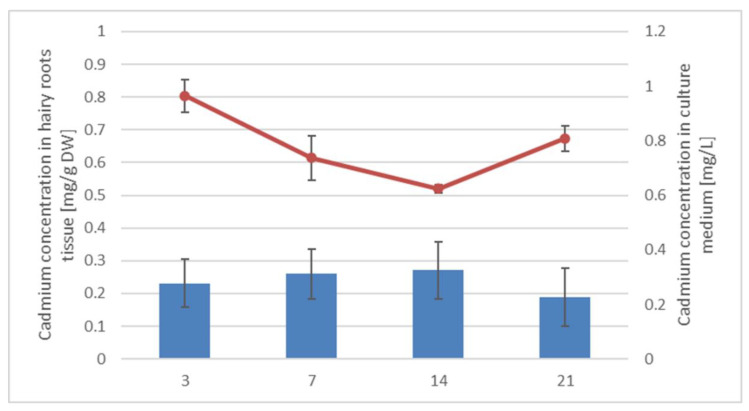
Cadmium concentrations in hairy roots tissue (blue bars) and culture medium (red curve). Values represent mean ± SD.

The cadmium concentrations in hairy roots tissue and culture medium were measured via the Flame Atomic Absorption Spectroscopy (FAAS) method as described in Section 4.11; the obtained results are presented in Figure 3. The Cd concentration in hairy root tissue gradually increased up to 14 days, whereas in the medium, it decreased over time. However, on the 21st day of the experiment, the cadmium concentration in hairy root tissue decreased by 30%, and the content of cadmium ions in the culture medium increased, most likely because of the disruption of root tissues and the release of accumulated cadmium ions into the culture medium.

#### 2.1.3. Effect of Cadmium on the Content of Steroids

**Figure 4:**
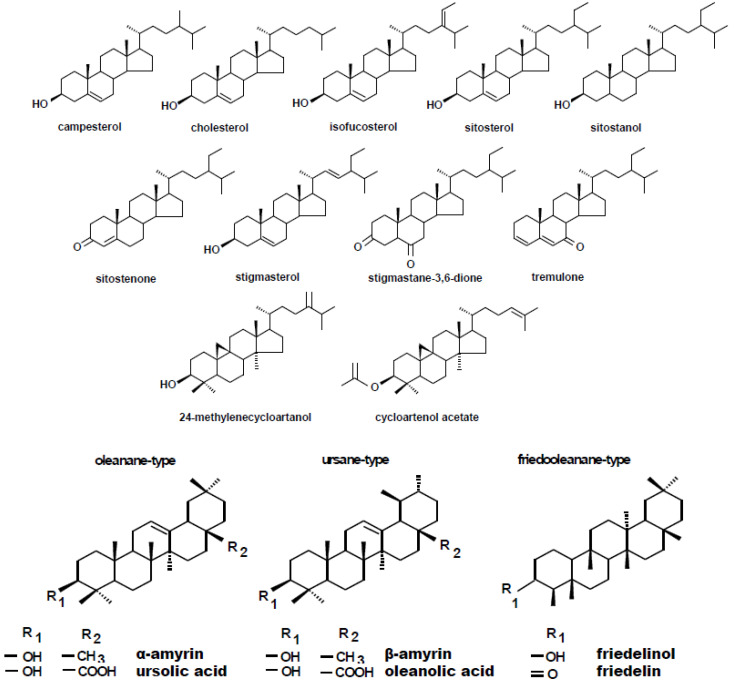
Chemical structures of compounds identified in hairy roots and native marigold plants described in this study.

The fraction of steroids in root tissue in the free form (not esterified or glycosylated) was obtained chromatographically from the diethyl ether extracts of hairy roots, as described in Section 4.4. Based on GC-MS analysis, the occurrence of a group of typical sterols, namely, campesterol (24*R*-ergost-5-en-3β-ol); cholesterol (cholest-5-en-3β-ol); isofucosterol (24*Z*-stigmasta-5,24(28)-dien-3β-ol, synonym: Δ5-avenasterol); sitosterol (stigmast-5-en-3β-ol), accompanied by its saturated form, sitostanol; and the predominating stigmasterol (22*E*-stigmasta-5,22-dien-3β-ol), was observed. One steroid ketone, tremulone (stigmasta-3,5-dien-7-one), and one biosynthetic precursor, 24-methylenecycloartanol (24-methylene-9,19-cyclo-9β-lanostan-3β-ol), were also identified in this fraction. Figure 4 presents the chemical structures of the identified compounds. All analyzed sterols and steroids were identified according to their MS spectra (Table S1); the results were supported by the comparison of their retention time and chromatographic mobility to the respective parameters of available authentic standards.

**Figure 5:**
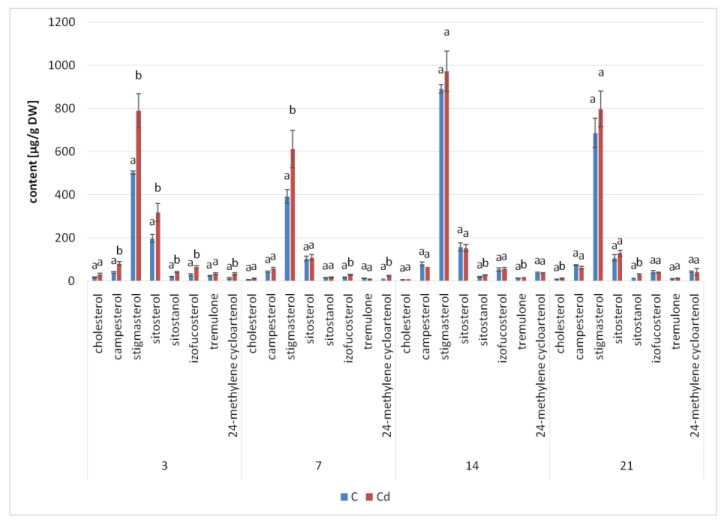
The effect of cadmium treatment on steroid content. Bars which do not share a common letter are significantly different (*p* < 0.05).

**Figure 6:**
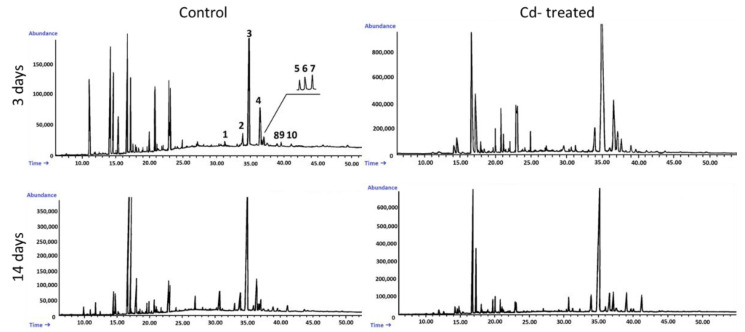
Representative chromatograms of the fractions containing free steroids from diethyl ether extracts obtained from control and cadmium-treated hairy roots after 3 and 14 days: 1, cholesterol; 2, campesterol; 3, stigmasterol; 4, sitosterol; 5, sitostanol; 6, isofucosterol; 7, β-amyrin; 8, α-amyrin; 9, tremulone; 10, 24-methylenecycloartanol.

The presence of cadmium ions in the culture medium caused a significant increase in steroid biosynthesis (Figure 5 and Figure 6, Table S2); the total contents of these compounds in Cd-treated hairy roots were significantly higher than those in untreated roots throughout the experiment. A particularly significant effect was noticed in samples collected after 3 and 7 days of incubation, when the increase of the steroid content in Cd-treated roots (as compared with untreated roots) was approximately 44% and 31%, respectively. Afterwards, this difference in the total steroid content between Cd-treated and control roots diminished, with a decrease to 7% and 12% in samples collected after 14 and 21 days of incubation, respectively.

Simultaneously, although the basic composition of steroids remained stable in both Cd-treated and control roots, changes in the proportions of the individual constituents could be observed. A particularly remarkable difference was noticed in the stigmasterol-to-sitosterol ratio after 7 days of incubation, which equaled 3.8:1 in control samples and 5.6:1 in Cd-treated samples, due to the significant increase in the stigmasterol content. However, in samples collected after 3, 14, and 21 days, the stigmasterol-to sitosterol-ratio was similar in Cd-treated and untreated roots.

Another characteristic phenomenon was the change in the content of campesterol, which had doubled in Cd-treated roots after 3 days of incubation. After 7 days of incubation, it was still slightly higher (by 26%) than in the control samples, whereas after both 14 and 21 days of incubation, its content in Cd-treated roots had markedly decreased and was 16% lower than that in the untreated samples. A similar tendency was noticed in the case of isofucosterol, whose content was two-fold higher in Cd-treated roots than in control samples after 3 days of incubation; after 7 and 14 days, it was still 41% and 7% higher, respectively, than that in untreated roots. Finally, after 21 days of incubation, it was 7% lower in Cd-treated roots than in the control samples. In turn, the content of 24-methylenecycloartanol, one of the sterol precursors in the biosynthetic pathway, was almost three-fold higher in Cd-treated roots than in untreated samples after 3 days of incubation. After 7 days of incubation, its content was still two-fold higher in Cd-treated roots, but subsequently, after 14 and 21 days of incubation, this difference vanished.

**Figure 7:**
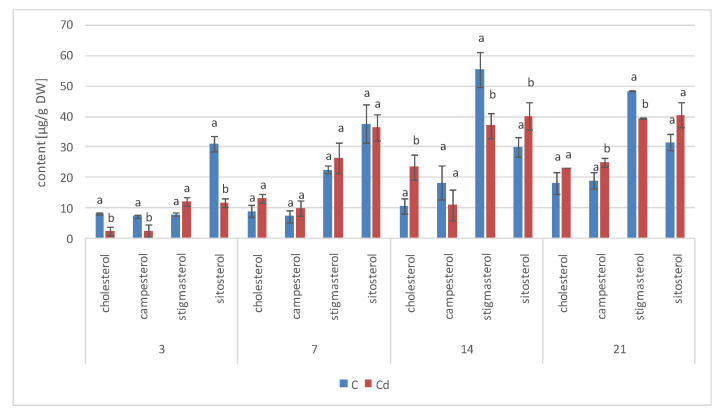
The effect of cadmium treatment on the content of sterol esters. Bars which do not share a common letter are significantly different (*p* < 0.05).

Four sterols, i.e., campesterol, cholesterol, sitosterol, and stigmasterol, were also found in ester and glycoside fractions of diethyl ether and methanol extracts subjected to hydrolysis (Section 4.5, Section 4.6 and Section 4.7, Tables S3 and S4). The content of sterols occurring in ester form decreased sharply (almost two-fold) in Cd-treated roots after 3 days of incubation (Figure 7). However, this effect did not last long, and after 7 days of incubation, the content of sterol esters had increased by 12% in Cd-treated samples. After 14 days of incubation, almost similar sterol ester contents were found in treated and untreated roots, whereas after 21 days of incubation, the levels were slightly higher in Cd-treated roots (by 9%). The proportions among individual sterols in the ester fraction were not identical, as in the case of their free forms. For example, stigmasterol, which prevailed among the free sterols (constituting approximately 60% of these compounds), was much less abundant in the ester fraction; its content increased (regarding the control samples) to exceed the content of sitosterol only after 14 and 21 days of the experiment. Moreover, in Cd-treated roots, stigmasterol was less abundant than sitosterol in all analyzed samples apart from those collected after 3 days of incubation, when the contents of these two sterols were almost similar.

**Figure 8:**
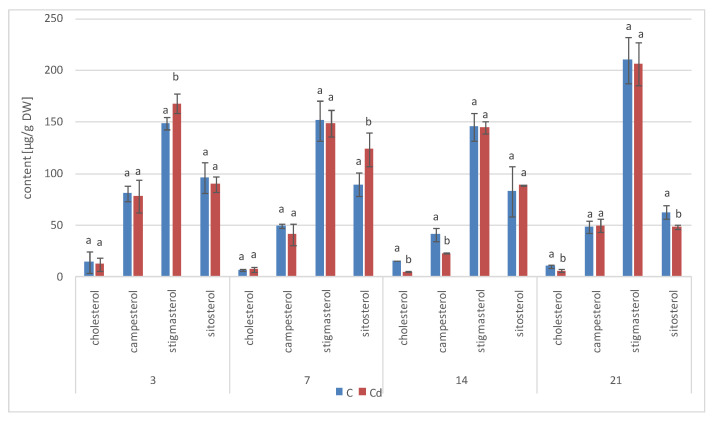
The effect of cadmium treatment on the content of sterol glycosides. Bars which do not share a common letter are significantly different (*p* < 0.05).

The contents of sterol glycosides were similar in control and Cd-treated roots after 3 days of incubation and subsequently increased by 7% after 7 days of the experiment. However, after a longer period of Cd exposure, these levels decreased, namely, by 8% and 6% after 14 and 21 days of incubation, respectively (Figure 8). The proportion of stigmasterol to sitosterol was modified first due to the slight increase of the content of stigmasterol (stigmasterol-to-sitosterol ratio equaled 1.6:1 in control samples and almost 2:1 in Cd-treated roots after 3 days of incubation) and due to the significant increase in the sitosterol level (1.7:1 in control samples versus 1.2:1 in Cd-treated roots). After 14 and 21 days of the experiment, the stigmasterol-to-sitosterol ratio was similar for control and Cd-treated roots. Another noticeable effect was the simultaneous decrease of the campesterol and cholesterol levels (by two-fold and three-fold, respectively) in Cd-treated roots after 14 days of incubation.

#### 2.1.4. Effect of Cadmium on the Content of Triterpenoids

**Figure 9:**
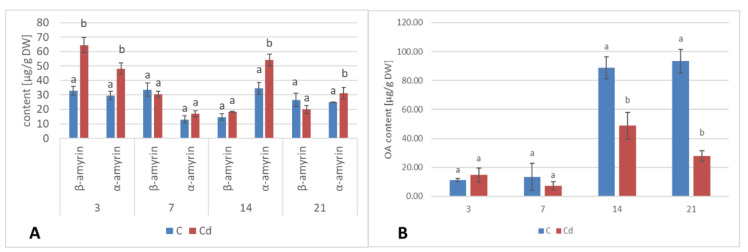
The effect of cadmium treatment on the content of neutral triterpenoids (amyrins) (**A**) and free oleanolic acid (OA) (**B**) in hairy roots tissue. Bars which do not share a common letter are significantly different (*p* < 0.05).

In diethyl ether extracts from root tissues, two triterpenoid alcohols were identified, namely, α- and β-amyrins (i.e., urs-12-en-3β-ol and olean-12-en-3β-ol), precursors of the triterpenoids of ursane and oleanane types of the skeleton. Their contents increased almost two-fold in Cd-treated roots after 3 days of incubation, followed by a decrease; after 7 days of incubation, the content of amyrins was 32% higher in Cd-treated samples compared to the control. With further incubation, control and treated samples showed similar levels (Figure 9A, Table S5). Simultaneously, the content of free oleanolic acid (3β-hydroxy-olean-12-en-28-oic acid) in Cd-treated roots also first slightly increased (by 26% after 3 days of incubation) and then sharply decreased (almost two-fold after both 7 and 14 days of incubation and more than three-fold after 21 days of incubation (Figure 9B, Table S6).

**Figure 10:**
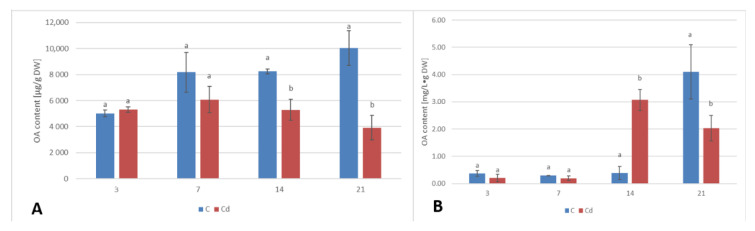
The effect of cadmium treatment on the content of saponins in the root tissue (**A**) and saponins released to the culture medium (**B**). Bars which do not share a common letter are significantly different (*p* < 0.05).

The content of saponins (glycosylated oleanolic acid) in root tissues and secreted into the culture medium was determined in methanol and butanol extracts, respectively, and expressed as the content of oleanolic acid released from its glycosides after acidic hydrolysis (as described in Section 4.3, Section 4.6, and Section 4.7). The content of saponins accumulated in the root tissue increased slightly (by 6%) after 3 days of incubation with Cd and then gradually decreased, with decreases by 26% and 36% after 7 and 14 days of the experiment, respectively, and finally sharply, up to more than 2.5-fold, after 21 days of incubation (Figure 10A, Table S7). In turn, the secretion of saponins into the medium from the Cd-treated roots decreased by approximately two-fold and by 37% in the root cultures exposed to Cd-treatment after 3 and 7 days of incubation, respectively, followed by a sharp increase, by eight-fold, after 14 days of the experiment. Subsequently, the level decreased again by two-fold in comparison with the control samples (Figure 10B, Table S8).

### 2.2. Cadmium Influence on C. officinalis Plants

**Figure 11:**
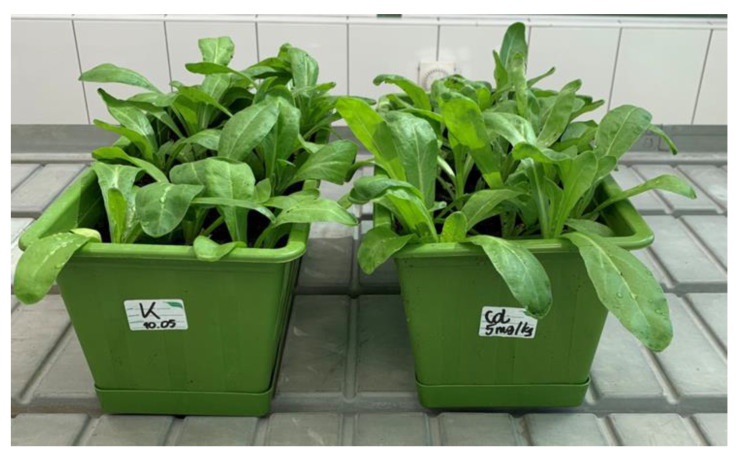
Three-week-old *C. officinalis* plants cultivated in the greenhouse.

*Calendula officinalis* plants (at the developmental stage of 3-week-old seedlings, Figure 11) were transferred to soil supplemented with cadmium ions as described in Section 4.2.2. Growth parameters and cadmium accumulation, as well as the contents of steroids and triterpenoids were determined separately in roots and aerial parts (shoots) of the plants collected after 7 and 14 days of growing in Cd-contaminated soil.

#### 2.2.1. Cadmium Effect on Root and Shoot Growth Parameters

**Figure 12:**
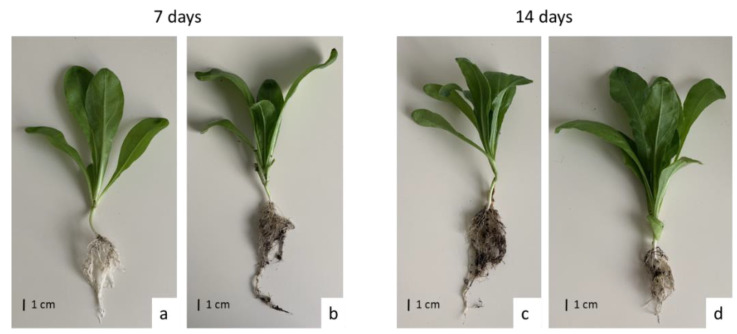
Control (**a**,**c**) and cadmium-treated (**b**,**d**) *C. officinalis* plants after 7 and 14 days of cultivation.

**Figure 13:**
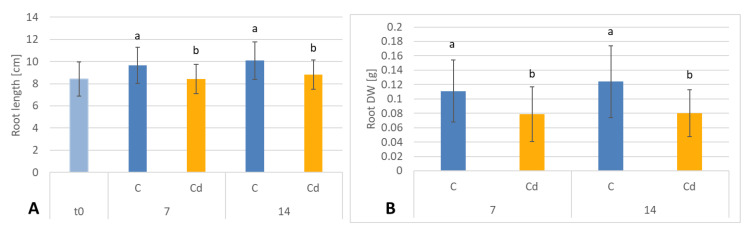
The length (**A**) and dry weight DW (**B**) of *C. officinalis* plant roots. t0 (light blue bar)- root length of three-week-old seedlings. Bars which do not share a common letter are significantly different (*p* < 0.05).

The presence of cadmium in the soil significantly influenced the growth of *C. officinalis* plants (Figure 12). Both after 7 and 14 days of cultivation in Cd-contaminated soil, the length of roots were approximately 13% shorter compared to those of untreated plants (Figure 13A). Growth inhibition also manifested itself in the decrease in root DW after 7 and 14 days of exposure to cadmium, namely, by 27% and 33%, respectively, compared to control plants (Figure 13B).

**Figure 14:**
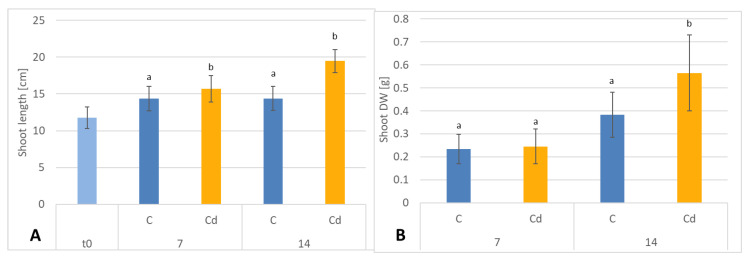
The length (**A**) and dry weight (**B**) DW of *C. officinalis* plant shoots. t0 (light blue bar)- shoot length of three-week-old seedlings. Bars which do not share a common letter are significantly different (*p* < 0.05).

In contrast, shoot length and shoot DW of plants cultivated in Cd-contaminated soil were higher than in control plants. In Cd-treated plants, the shoots were 9% and 26% longer after 7 and 14 days of cultivation, respectively, than the shoots of untreated plants (Figure 14A). The corresponding shoot DW was higher in shoots of Cd-treated plants than in control plants, namely, by 8% and 32% % after 7 and 14 days of cultivation, respectively (Figure 14B).

#### 2.2.2. Accumulation of Cadmium in Roots and Shoots

**Figure 15:**
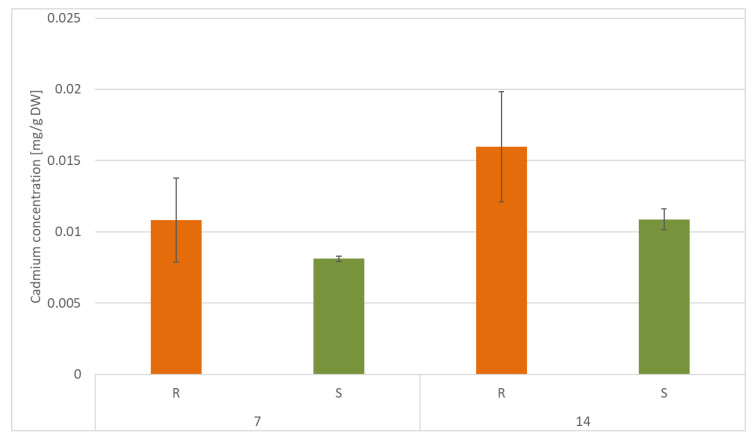
Cadmium concentration in roots (R) and shoots (S) of *C. officinalis* plants after 7 and 14 days of cultivation in Cd- contaminated soil. Values represent mean ± SD.

According to the data obtained from FAAS analysis, the content of Cd was significantly higher in roots than in shoots, namely, by 25% and 32% after 7 and 14 days of cultivation, respectively. However, in both roots and shoots, Cd accumulation decreased during cultivation in Cd-contaminated soil, namely, by 32% in roots and by 25% in shoots (Figure 15).

The translocation factor (TF) for Cd-treated samples, calculated as the ratio of the Cd concentration in roots to that in shoots, was above 1 for both 7 and 14 days, indicating the transfer of Cd from the roots to the shoots of the plants.

#### 2.2.3. Effect of Cadmium on the Content of Steroids in *C. officinalis* Roots

**Figure 16:**
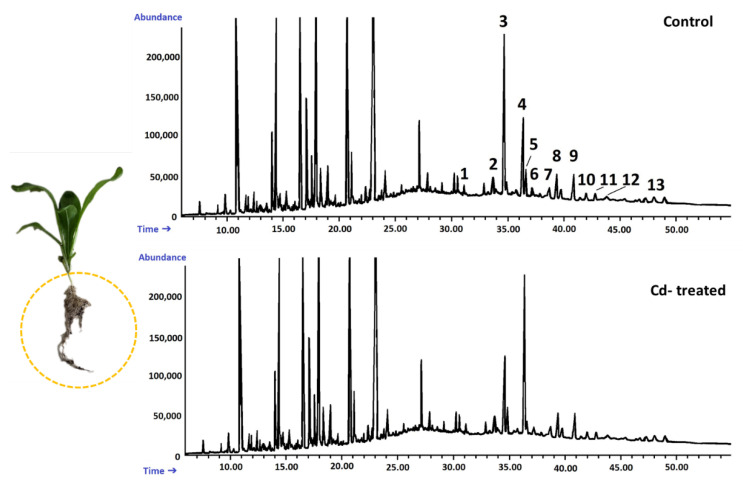
Representative chromatograms of the fractions containing free steroids from diethyl ether extracts obtained from control and cadmium-treated roots of marigold plants after 14 days of cultivation: 1, cholesterol; 2, campesterol; 3, stigmasterol; 4, sitosterol; 5, sitostanol; 6, β-amyrin; 7, α-amyrin; 8, tremulone; 9, sitostenone; 10, cycloartenol acetate; 11, friedelinol; 12, friedelin; 13, stigmastane-3,6-dione.

According to GC-MS analysis, the profile of steroids in *C. officinalis* roots consisted of several compounds previously identified in hairy roots, i.e., campesterol, cholesterol, sitosterol accompanied by sitostanol, the predominating stigmasterol, and a steroid ketone, tremulone (Figure 16). Thus, isofucosterol and 24-methylenecycloartanol were not found in the roots of *C. officinalis* plants, at least in detectable amounts, but instead, two additional steroid ketones, sitostenone (stigmast-4-en-3-one) and stigmastan-3,6-dione (the latter only in older roots), as well as one derivative of sterol precursor, cycloartenol acetate, were identified.

**Figure 17:**
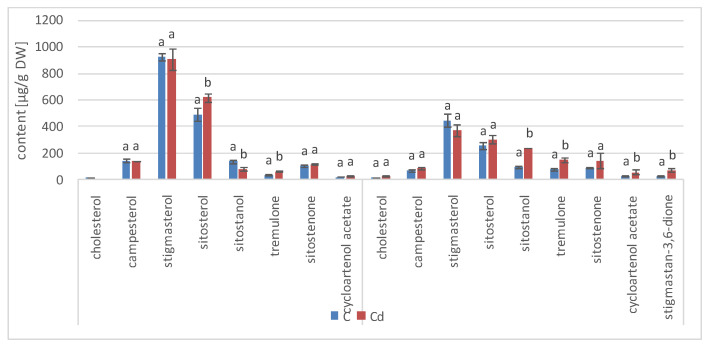
The content of free sterols in roots of control and Cd- treated *C. officinalis* plants after 7 and 14 days of cultivation. Bars which do not share a common letter are significantly different (*p* < 0.05).

As observed previously for hairy root cultures, the total content of steroids in roots initially increased in *C. officinalis* plants exposed to Cd stress (after 7 days of cultivation in Cd-contaminated soil) only by 4%; however, after 14 days of the experiment, it had increased by 30% (Figure 17, Table S9). Simultaneously, the stigmasterol-to-sitosterol ratio changed due to the increased content of sitosterol; after 7 days of incubation, this ratio was 1.9:1 in control samples and 1.7:1 in Cd-treated samples, whereas after 14 days of incubation, this difference was even more pronounced: 1.5:1 in control samples and 1.2:1 in Cd-treated samples.

Noticeable changes in the contents of other compounds appeared after 14 days of cultivation in Cd-contaminated soil. For example, the content of the saturated sterol form, sitostanol, was 2.5-times higher in Cd-treated roots; the amounts of steroid ketones, sitostenone and stigmastan-3,6-dione, were also significantly increased (by 37% and more than three-fold, respectively). In contrast, the content of campesterol was not significantly increased after Cd-treatment. Cholesterol was not detected in Cd-treated roots after 7 days of cultivation, and it appeared in samples collected after 14 days of the experiments in amounts slightly higher than those of the control samples.

**Figure 18:**
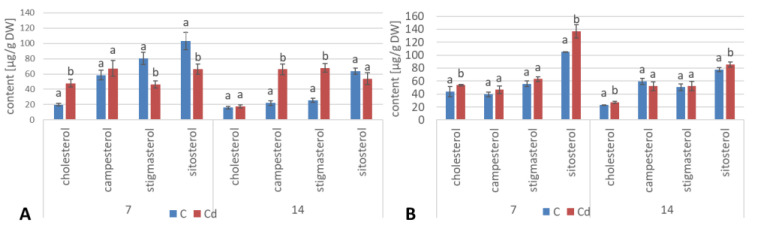
The content of sterol esters (**A**) and sterol glycosides (**B**) in *C. officinalis* roots. Bars which do not share a common letter are significantly different (*p* < 0.05).

As in the hairy root cultures, four sterols, i.e., campesterol, cholesterol, sitosterol, and stigmasterol, were found in *C. officinalis* plant roots in the form of esters and glycosides. The content of sterols occurring in the ester form decreased slightly (by 13%) after 7 days of cultivation in Cd-contaminated soil; subsequently, after 14 days of the experiment, it increased by 38% as compared with the control samples. As observed for hairy roots, the proportions among individual sterols in the ester fraction were not identical as in the case of their free forms; again, sitosterol (and not stigmasterol) was the most abundant ester form of sterols in control samples (Figure 18A, Table S10). However, the content of stigmasterol increased significantly in roots after 14 days of Cd exposure, slightly exceeding that of sitosterol. The content of campesterol in the ester form was higher in roots of Cd-treated plants, particularly after 14 days of Cd exposure, when it increased three-fold as compared to the control samples.

The contents of sterol glycosides in roots of plants cultivated in Cd-contaminated soil increased significantly by 18% after 7 days of the experiment and subsequently returned to the control level after 14 days of the experiment (Figure 18B, Table S10). As in the ester fraction, sitosterol was the predominating sterol. The proportion of stigmasterol to sitosterol in the fraction of sterol glycosides was inverted due to the higher content of sitosterol; it equaled 1:2.3 and 1:2.2 in control and Cd-treated samples, respectively, after 7 days of the experiment, and 1:1.5 and 1:1.6 after 14 days of the experiment.

#### 2.2.4. Effect of Cadmium on the Content of Steroids in *C. officinalis* Shoots

**Figure 19:**
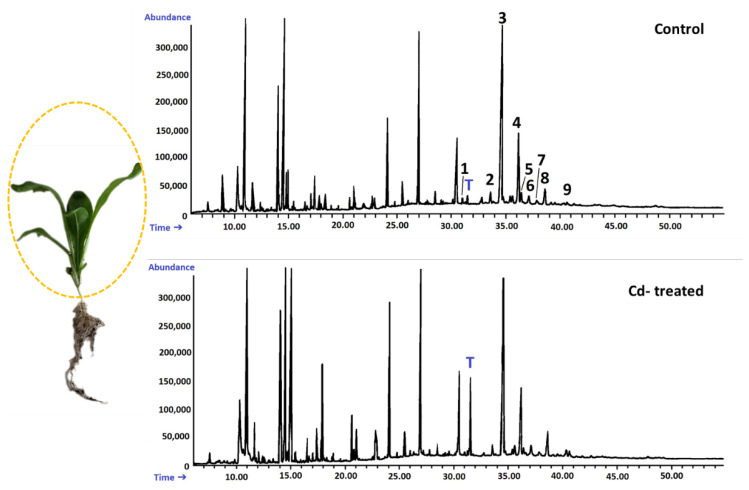
Representative chromatograms of the fractions containing free steroids from diethyl ether extracts obtained from control and cadmium-treated shoots of marigold plants after 14 days of cultivation: 1, cholesterol; 2, campesterol; 3, stigmasterol; 4, sitosterol; 5, sitostanol; 6, β-amyrin; 7, α-amyrin; 8, tremulone; 9, sitostenone. T—α-tocopherol.

**Figure 20:**
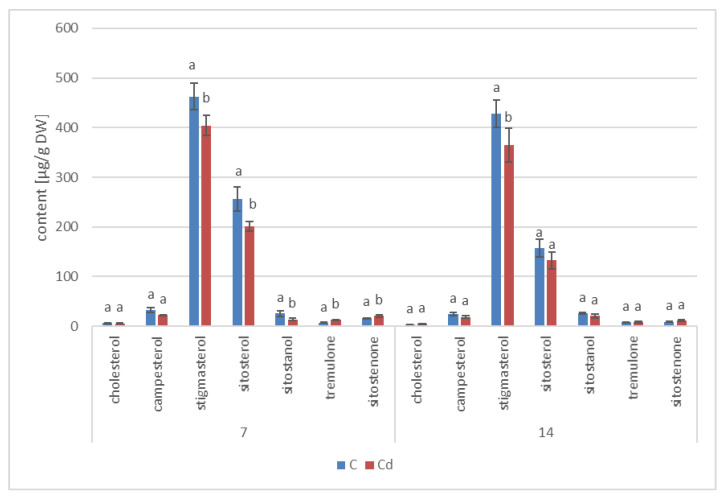
Free sterols content in shoots of control and cadmium- treated marigold plants after 7 and 14 days of cultivation. Bars which do not share a common letter are significantly different (*p* < 0.05).

The profile of steroids occurring in a free form in extracts from *C. officinalis* shoots consisted of the group of typical sterols, campesterol, cholesterol, sitosterol (accompanied by sitostanol), the predominating stigmasterol, and two steroid ketones, tremulone and sitostenone (Figure 19). In contrast to the effects observed in roots and previously in the hairy root culture, the total content of steroids slightly decreased in samples collected from the plants cultivated in Cd-contaminated soil, namely, by approximately 15% after both 7 and 14 days of Cd exposure (Figure 20, Table S11). The amounts of the two most abundant sterols, i.e., stigmasterol and sitosterol, were particularly influenced, with the content of stigmasterol decreasing by 13% and 15% after 7 and 14 days of the experiment, respectively; the content of sitosterol decreased by 22% and 16% after the respective durations of Cd exposure. However, the stigmasterol-to-sitosterol ratio was not significantly changed; the most pronounced difference was noticed after 7 days of the experiment, when this proportion increased from 1.8:1 in the control samples to 2:1 in the shoots of Cd-treated plants. The only compounds of the free steroid fraction in shoots whose contents increased during Cd-treatment were the steroid ketones tremulone (by 46% and 20% after 7 and 14 days of cultivation in Cd-contaminated soil, respectively) and sitostenone (by 8% and 18% after 7 and 14 days of the experiment, respectively).

**Figure 21:**
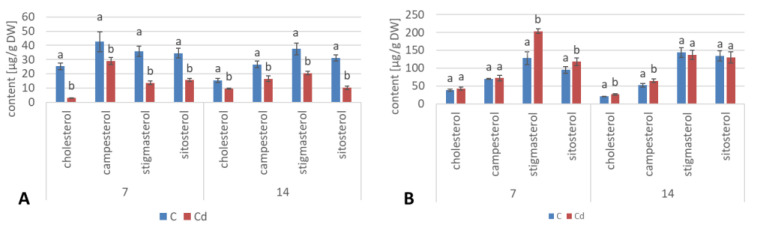
The content of sterol esters (**A**)and sterol glycosides (**B**) in *C. officinalis* shoots. Bars which do not share a common letter are significantly different (*p* < 0.05).

The ester fraction constituted the minor fraction in *C. officinalis* shoots. Its content decreased significantly, by more than two-fold both after 7 and 14 days the experiment (Figure 21A, Table S12). As in the case of *C. officinalis* roots and hairy root culture, the proportions among individual compounds occurring in the ester form differed from that among free sterols, particularly in samples collected after 7 days of cultivation, when campesterol was the most abundant sterol both in control samples and in shoots of plants exposed to Cd stress; stigmasterol and sitosterol occurred in comparable amounts. However, in older shoots, stigmasterol was again the prevailing compound in the ester fraction, as it was among free sterols.

The content of sterol glycosides in shoots of plants cultivated in Cd-contaminated soil increased significantly by 24% after 7 days of the experiment and subsequently returned to the control level after 14 days of the experiment (Figure 21B, Table S12), as noticed in the roots. However, in this fraction, stigmasterol was the predominating sterol, although the proportion of stigmasterol to sitosterol changed during the experiment; it equaled 1.3:1 and 1.7:1 in control and Cd-treated samples, respectively, after 7 days of the experiment and 1.1:1 and 1.2:1 after 14 days of the experiment because of the increase in the sitosterol content.

#### 2.2.5. Effect of Cadmium on the Content of Triterpenoids in *C. officinalis* Roots and Shoots

**Figure 22:**
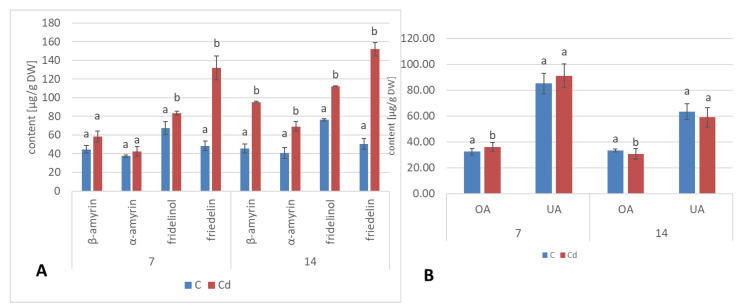
The content of neutral triterpenoids (**A**) triterpenoid acids (**B**) and in *C. officinalis* roots. OA—oleanolic acid, UA—ursolic acid. Bars which do not share a common letter are significantly different (*p* < 0.05).

**Figure 23:**
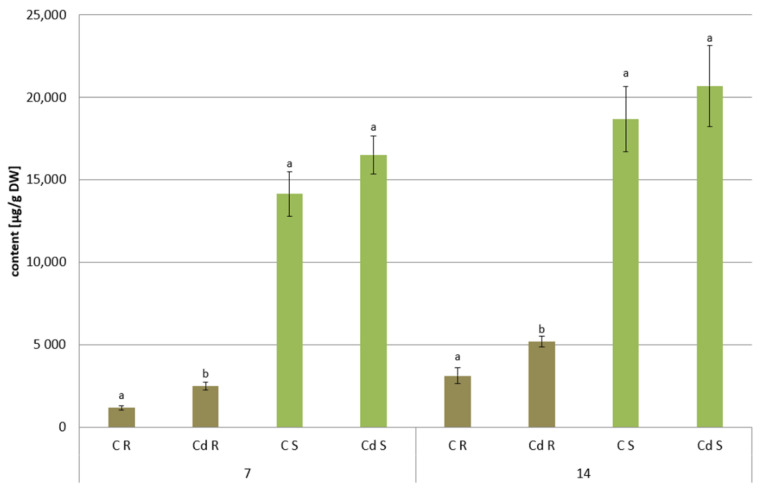
The content of saponins in *C. officinalis* roots (R) and shoots (S). Bars which do not share a common letter are significantly different (*p* < 0.05).

Apart from two triterpenoid alcohols, i.e., α- and β-amyrins, found in the hairy root culture, two other triterpenoids were identified in roots of *C. officinalis* plants, namely, friedelinol (D:A-friedooleanan-3α-ol) and friedelin (D:A-friedoolanan-3-one). The total content of the neutral triterpenoids (i.e., alcohols and ketones) significantly increased in roots exposed to Cd-stress by 37% after 7 days of cultivation in Cd-contaminated soil and two-fold after 14 days (Figure 22A, Table S9). In both control and Cd-treated roots, β-amyrin predominated over α-amyrin, friedelinol was more abundant in roots of the control samples and friedelin in Cd-treated roots. Moreover, free oleanolic acid, and its isomer, ursolic acid (3β-hydroxy-urs-12-en-28-oic acid), was identified in roots of *C. officinalis* plants. The contents of these two acids slightly increased (by 8%) in roots after 7 days of cultivation in Cd-contaminated soil and then slightly decreased (by 7%) after 14 days of the experiment (Figure 22B). In turn, the contents of saponins (exclusively glycosides of oleanolic acid; no glycosides of ursolic acid were detected) increased significantly in roots of Cd-treated plants, namely, two-fold and by 40% after 7 and 14 days of the experiment, respectively (Figure 23, Table S13).

**Figure 24:**
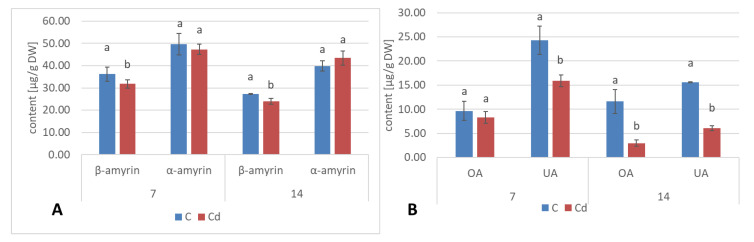
The content of neutral triterpenoids (**A**) and triterpenoid acids (**B**) in *C. officinalis* shoots. OA—oleanolic acid, UA—ursolic acid. Bars which do not share a common letter are significantly different (*p* < 0.05).

In shoots of *C. officinalis* plants, only two amyrins were detected in the fraction of the neutral triterpenoids. Their contents decreased slightly (by 8%) in shoots of plants exposed to Cd stress after 7 days of treatment and reached control levels after 14 days (Figure 24A). The contents of two isomeric acids, oleanolic and ursolic, decreased significantly in shoots of Cd-treated plants, namely, by 27% and three-fold after 7 and 14 days of cultivation in Cd-contaminated soil, respectively (Figure 24B). As in the roots, the contents of oleanolic acid glycosides increased in the shoots of Cd-treated plants by 24% and by 13% after 7 and 14 days of the experiment, respectively (Figure 23). It should be noticed that the content of saponins in shoots was several times higher than that in roots of *C. officinalis* plants.

A peak of retention time of 31.5 min, clearly visible on the GC chromatogram of the fractions of free steroids and triterpenoids from shoot extracts (Figure 19), was found to be associated with α-tocopherol. The content of this compound, calculated according to peak area, increased by three-fold in shoots of Cd-treated plants as compared with the control samples.

## 3. Discussion

This study investigated the influence of Cd stress on the steroid and triterpenoid metabolism in *C. officinalis* plants and hairy roots cultures and represents a targeted GC-MS metabolomic approach to study stress-induced metabolic modifications. As it has been frequently reported for toxic metals and metalloids, high Cd concentrations in plants can alter metabolic processes and exert various effects at the physiological, morphological, and molecular levels, e.g., growth inhibition, nutrition imbalance, photosynthesis suppression, chlorosis, and ROS (reactive oxygen species) increase [21,22]. Various reports indicate that the composition of steroids and triterpenoids might be modified under stress conditions; however, detailed studies on such effects exerted after exposure to heavy metals are scarce [11,23,24]. The first reported studies on this phenomenon, performed either on algae or plants, demonstrated effects such as increased levels of cholesterol and a decrease in the ratio of 24-ethylcholest-5-en-3β-ol to 24-ethylcholesta-5,24(28)*Z*-dien-3β-ol, two major sterols occurring in the marine diatom *Asterionella gracilis* [25], or the increase of stigmasterol and sitosterol contents parallel to the decrease of isofucosterol and campesterol, as observed for rice (*Oryza sativa* cv. Bahía) seedlings [26,27] exposed to Cd stress.

The results obtained in the present study clearly indicate that Cd stress significantly altered the contents of steroids, including sterols, as well as the proportions among individual constituents. However, it was observed that various organs of the same plant can react differently to Cd stress. The total sterol content increased in roots and hairy root culture and decreased in shoots; these effects were inversely correlated with Cd-induced growth suppression. A lack of a direct correlation between the changes in the growth and total sterol content has also been observed in previous studies, e.g., on *O. sativa* plants exposed to Cd stress, where the reduced growth of both shoots and roots was accompanied with an approximately 30% increase in the sterol content. The growth suppress parallel to the stimulation of sterol biosynthesis can be explained by the restructuring of the membrane composition due to the accumulation of the excess sterols, leading to substantial changes in membrane properties, such as fluidity, permeability, and perception of stress signals; such effects have frequently been observed in plants and in particular in in-vitro cultures exposed to various stressors or elicitors [20,28]. Regarding the essential role of sterols as plasma membrane constituents, the explanation of the adverse phenomenon, i.e., the correlation between increased growth and a decrease in the sterol content, seems to be less obvious; however, the increase in biomass of plants and in-vitro cultures might not occur exclusively as a result of the increased number of cells. Indeed, in a previous study, the reported increase in the dry weight of mercury-treated algal cells appeared to be uncoupled from cell division [25]. Moreover, the inhibition of sterol biosynthesis, according to the “plant Cornelian dilemma” of the growth-defense trade-off [24], can significantly induce the biosynthesis of triterpenoids or other compounds excessively accumulating in cells under stress conditions

The observed decrease in both *C. officinalis* plant roots and hairy root culture growth is in accordance with most of the studies on heavy metal influences on plant development. The reduction of the biomass in plants and plant in vitro cultures exposed to Cd stress can be attributed to the accumulation of this element in the cell wall and its entry into the cytoplasm, leading to the disruption of the normal metabolism, the reduction of the uptake and distribution of other essential elements and nutrients (thus inducing mineral deficiencies and interrupting the activity of important enzymes), and reduction in the cellular turgor and cell division. Plant roots are the first organs directly affected by heavy metals present in soil, and the exerted phytotoxic effects are clearly visible as root growth inhibition and alterations in root morphology. Roots are particularly affected by Cd-induced stress, because Cd can be readily absorbed into the rhizodermis and root cortex, either through apoplastic or symplastic pathways, and then through the plasma membrane of the endodermis, root cell walls are the major sites of Cd storage, and Cd accumulation is, therefore, generally greater in roots compared with the plant’s aerial parts [22,29]. The present study clearly shows that in *C. officinalis* plants, the root is a direct target of Cd stress and is more affected than the shoot. Furthermore, the aerial part of the plants exposed to Cd might not display symptoms of growth reduction; on the contrary, both the biomass and the height of shoots of plants cultivated in Cd-contaminated soil were greater than those of untreated plants. Similar observations have been reported for *C. officinalis* exposed to Cd stress, indicating that relatively low Cd concentrations in the soil could stimulate plant growth [18]. This phenomenon has also been reported in other studies and has been termed “hormesis” by Jia et al. (2015) [30]. The present study confirms earlier findings that *C. officinalis* plants show high tolerance to Cd contamination; as this species is largely used as an ornamental plant and therefore does not reach the human food chain, it might be used for phytoremediation purposes [18,19]. However, this implies the distinct understanding that it should not be harvested for other purposes, e.g., as a herbal plant in traditional medicine.

Regardless of the Cd influence on the total sterol content, the present study revealed that Cd stress affected the proportions among individual compounds and the formation of ester and glycoside conjugates. The main observations concerned the changing ratio between stigmasterol and sitosterol, resulting from modulations of sterol side-chain hydrogenation as well as fluctuations in the campesterol content, indicating the diverse stimulation of competing branches in sterol biosynthesis, i.e., 24-methyl sterols versus 24-ethyl sterols. Changes in the sitosterol-to-stigmasterol ratio are frequently observed in plants during adaptation to unfavorable environmental conditions, e.g., under drought stress or salinity [11,12]. Stigmasterol, formed from sitosterol in a reaction catalyzed by C-22-sterol desaturase, has an additional double bond in the side chain and can induce substantial changes in the properties of cell membranes correlated with altered activities of membrane-associated enzymes and activation of proton pumps such as H^+^-ATPases, the including PM (plasma membrane) proton pump, which are responsible for pH regulation and the production of gradients necessary to maintain cellular homeostasis [31]. It has been suggested that sterols improve the resistance of plants through reinforcing the cohesion of the cell membrane and may preserve the active conformation of PM H^+^-ATPase, modulating its interaction with other constituents, e.g., phospholipids. Although the modulation of PM H^+^-ATPase by sterols might be a rather “slow response” because of the complexity of the sterol metabolism, it could nevertheless represent an important mechanism of adaptation to stress conditions [12,23]. Ester and glycoside conjugates of sterols are also considered to play a key role in maintaining cell membrane homeostasis, although their correlation with particular stress responses is not always evident [11]. In the present study, the increase in the content of glycoside conjugates and the decrease of ester forms were noticed, both in roots and hairy root culture, as well as in shoots. However, this phenomenon might not be universal for all plants and requires further studies. Moreover, it is still difficult to ascertain whether the observed changes in the content of sterols and their conjugates appear as a direct effect of Cd toxicity, and, thus, they constitute the primary mechanism of the response to Cd stress; or they are generated as a secondary effect of an interference with other metabolic pathways and modified activity of respective enzymes.

The obtained results clearly demonstrate that the metabolic modifications occurring during stress responses are not constant and equally intensive in time. Some of the observed phenomena could even have an inverse course, first increasing than decreasing, which was particularly evident during a relatively long experiment performed on the hairy root culture. This observation is important to understand that the time-course of plant stress responses is not uniform, the metabolism is a net of pathways influencing each other, and every change can have further consequences for the regulation of the biosynthesis of other compounds. The present study indicates that the majority of the observed modifications finally seem to tend to return to the normal physiological levels, e.g., the sharp boost of sterol biosynthesis observed in plant roots and hairy root culture decreased in time, and the differences between the Cd-treated and control samples largely decreased. However, it should be clearly noticed that the time-course and intensity of stress response differed in whole plants and hairy root cultures; usually the metabolic fluctuations appeared earlier in hairy roots, and were more instantaneous and profound.

A common phenomenon observed in plants subjected to stress conditions is the competitiveness of the primary/general and secondary/specialized metabolic pathways, including those of steroids and triterpenoids with a common precursor, namely, squalene [24]. Some symptoms of this competition could be observed in the present study, i.e., the increase in sterol biosynthesis parallel to the decrease in the triterpenoid content in *C. officinalis* plant roots and hairy root culture, as well as the inverse phenomenon, namely, the decrease in the sterol content accompanied by the stimulation of triterpenoid biosynthesis in shoots. Effects such as the significant increase in sterol content, changes in the proportion of stigmasterol to sitosterol, and a decrease in the biosynthesis of free oleanolic acid (however, it should be noted that the decreased level of oleanolic acid was coupled with a simultaneous increase in saponin secretion) have also been observed in a preliminary study on the possibility of the application of heavy metals as elicitors for the enhancement of the triterpenoid productivity in *C. officinalis* hairy root cultures [20]. In this sense, the obtained results suggest that sterols and their forms seem to play a greater role in the response to stress associated with the presence of Cd ions than specialized triterpenoid compounds, particularly in roots. This hypothesis is supported by the fact that the changes in sterol content, composition, and proportion between conjugated forms may affect the activity of H^+^-ATPases (regardless the mechanism of this modification, i.e., whether it occurs by a direct interaction of sterols with the proton pump, or by indirect alteration of the physical properties of the lipid bilayer), and, hence, influence the membrane transport and ion homeostasis, the processes essential for root cells. In turn, the increased level of α-tocopherol in shoots suggests that an enhanced synthesis of antioxidants is an important mechanism involved in a defensive response to Cd stress in aerial part of the plant.

Another aspect of the present study was the questioned suitability of hairy root cultures as an experimental model to investigate the mechanisms of plant stress responses. Heavy metals including Cd have been applied as efficient elicitors to enhance the biotechnological production of various valuable phytochemicals in plant hairy root cultures, e.g., phytoestrogenic isoflavones daidzein and genistein in *Psoralea corylifolia* [32] or ajmalicine in *Catharanthus roseus* [33]. However, various types of plant in vitro cultures were also often considered as useful experimental models for physiological research, particularly when the main aim was to examine the mechanisms and metabolic responses of plants rather than their agronomic characteristics. Experimental conditions in plant in vitro cultures can be more easily controlled than for soil-growing plants; the relative homogeneity of cultured cells or organs compared with whole plants (in plus to variability between individual plants) improves the reproducibility of results; additionally, the time required for experimental investigations is usually reduced [34,35]. The similarity of the metabolic modifications observed in the present study in *C. officinalis* plant roots and hairy root culture seems to be encouraging; however, as it has been frequently discussed in various reports, it is important to recognize the limitations associated with the use of in vitro study systems and to be careful before extrapolating the obtained results to plants under field conditions.

## 4. Materials and Methods

### 4.1. Plant Material

#### 4.1.1. Hairy Root Cultures

*C. officinalis* hairy roots line CC16 (derived from cotyledon explant) was obtained according to a previously described procedure [36]. The roots were cultivated in a ½ Murashige–Skoog liquid medium, at 23–25 °C, in the darkness on a rotary shaker at 120 rpm. Subcultures were performed every 3–4 weeks by transferring the 1–2 cm pieces of the young-branched root to 100 mL of a fresh medium.

#### 4.1.2. Pot Cultures

*C. officinalis* seeds (PNOS, Ożarów Mazowiecki, Poland) were sown into rectangular plastic pots (dimensions: 80 × 18 × 14 cm) filled with universal flower soil “Athena” (the details of physical and chemical characterization in Table S14). Germination of seed started within 3 days of sowing. The plants were cultivated for 3 weeks in the greenhouse under controlled conditions (16/8 h day/night fotoperiod, 52 ± 2% humidity, temperature 20 °C with a light intensity of 120 ± 10 µmol/m^2^·s).

### 4.2. Treatment with Cadmium Ions

#### 4.2.1. Supplementation of Hairy Roots

Freshly subcultured roots were incubated for 21 days to obtain at least 1.5 g of fresh weight. Afterwards, they were weighed and transferred to 100 mL of fresh medium five days prior to elicitation. A 50 mM stock solution of CaCl_2_ was prepared, then the solution was sterilized by filtration through a 0.22 m syringe filter (Millipore, Bionovo, Legnica, Poland) and added to the culture medium to obtain the final concentrations of 25 µmol/L. The cultures were cultivated with cadmium ions for 3, 7, 14, and 21 days.

#### 4.2.2. Supplementation of Pot-Cultures

Soil conditioning with cadmium was conducted by the addition of cadmium chloride salt solution (CaCl_2_), so that the final concentration of this element in soil was 5 mg Cd/kg dry weight [d.w]. After cadmium contamination, the soil was thoroughly mixed and incubated for 7 days at room temperature. During this time, the soil was mixed and watered every day in order to keep the humidity of the soil stable at 40–45%. Three-week-old seedlings were transferred to cadmium-contaminated soil and cultivated either for 1 week or for 2 weeks in the greenhouse under conditions described above.

### 4.3. Extraction and Fractionation

#### 4.3.1. Extraction of the Hairy Roots and the Culture Medium

After 3, 7, 14, and 21 days of incubation with cadmium, the culture media were filtered from the hairy roots. The harvested hairy roots were air-dried at room temperature before extraction, whereas the culture media were directly extracted 3 times with 40 mL portions of n-butanol to extract oleanolic acid saponins released to the medium. Dried hairy roots were powdered and extracted using a Soxhlet apparatus for 8 h with diethyl ether to obtain fractions of free sterols, conjugated sterols (esters and low polar glycosides), free triterpenoid acids and alcohols, and then 8 h with methanol to extract more polar sterol glycosides and oleanolic acid saponins. The obtained extracts were evaporated to dryness under reduced pressure on a rotary evaporator.

#### 4.3.2. Extraction of the Plants

After 7 and 14 days of growing in cadmium-contaminated soil, plants were gently collected, then weighed, and lengths of the root and the shoots (aerial) parts were measured. Roots and aerial parts were left to dry in the dark and airy place, afterwards weighed again, grinded in mortar to fine powder and extracted in Soxhlet apparatus for 8 h with diethyl ether and then 8 h with methanol. The obtained extracts were evaporated to dryness under reduced pressure on a rotary evaporator.

### 4.4. Fractionation of Diethyl Ether Extracts

Evaporated diethyl ether extracts obtained from the hairy roots, as well as roots and aerial parts of native plants, were fractionated by adsorption preparative TLC on 20 cm × 20 cm glass plates coated manually with silica gel 60H (Merck, Darmstadt, Germany). The solvent system chloroform: methanol 97:3 (*v*/*v*) was applied for developing. Four fractions were obtained as described by Sykłowska-Baranek et al. (2022): (i) esters (Rf, retention factor, of 0.9–1); (ii) free steroids and neutral triterpenoids (Rf of 0.3–0.9); (iii) free triterpenoid acids (Rf 0.2–0.3); and (iv) glycosides (Rf of 0–0.2) [28]. Fraction (ii) containing free steroids and neutral triterpenoids (alcohols) was directly analyzed using GC–MS, gas chromatography-mass spectrometer (Agilent Technologies 7890A); the triterpenoid acid fraction (iii) was methylated with diazomethane prior to GC-MS analysis as described previously [37]; the ester fraction (i) was subjected to alkaline hydrolysis to release the steroid core from ester forms; and the glycoside fraction (iv) to acidic hydrolysis to release the aglycones.

### 4.5. Alkaline Hydrolysis

The ester fractions were subjected to alkaline hydrolysis with 10% NaOH in 80% methanol at 80 °C for 3 h. Subsequently, 5 volumes of water were added to each hydrolysate, the pH was neutralized with 5% acetic acid, and the obtained mixtures were extracted with diethyl ether (3 × 20 mL) in a separation funnel. The extracts were evaporated and fractionated by preparative TLC (as described in Section 4.4) to obtain the fraction of sterols released from their ester forms.

### 4.6. Acidic Hydrolysis

The glycoside fractions obtained from the diethyl ether extracts, evaporated methanol extracts and n-butanol extracts from the culture medium were hydrolyzed by 11% HCl in 80% methanol during 2 h on a heating mantle under reflux [28,37]. Subsequently, the hydrolysates were diluted with distilled water, methanol was evaporated in a rotary evaporator, and the obtained aqueous remnants were extracted 3 times with 40 mL portions of diethyl ether in a separation funnel. The obtained extracts were washed with distilled water 3 times and evaporated to dryness.

### 4.7. Fractionation of Acidic Hydrolysates

The dried extracts obtained from acidic hydrolysates were divided by preparative TLC on 20 cm × 20 cm glass plates, manually coated with silica gel 60H (Merck, Darmstadt, Germany). The solvent system chloroform: methanol 95:5 (*v*/*v*) was used for developing the plates. From hydrolyzed methanol extracts, two fractions were obtained: sterols and oleanolic acid. From hydrolyzed n-butanol extracts, only one fraction was obtained: oleanolic acid. Purified oleanolic acid was methylated with diazomethane.

### 4.8. Derivatization of Triterpenoid Acids

Nitrosomethylurea (2.06 g) was added to a mixture of 20 mL of diethyl ether and 6 mL of 50% aqueous KOH, and the organic layer was then separated from the aqueous layer. Samples containing triterpenoid acids were dissolved in 2 mL of the obtained solution of diazomethane in diethyl ether, and held at 2 °C for 24 h.

### 4.9. Quantification of Oleanolic Acid by GC

The samples were solved in the suitable portion of the mixture diethyl ether: methanol 3:1 (*v*/*v*). Quantitative measurement of oleanolic acid (in the form of methyl ester) was performed by gas-liquid chromatography (GLC) at 270 °C on a Shimadzu GC-2014 instrument equipped with a flame ionization detector. Samples were applied by split injection 1:5 on a ZB-1 30 m × 0.25 mm × 0.25 μm column (Phenomenex, Torrance, CA, USA). The temperature of the injector and detector was 290 °C. Nitrogen was used as the carrier gas at a flow rate of 1.2 mL/min. Peak identification and quantification of oleanolic acid were carried out by referring to a calibration curve prepared with an authenticated sample of methylated oleanolic acid as the standard [38].

### 4.10. Identification and Quantification of Triterpenoids by GC–MS/FID

An Agilent Technologies 7890 A gas chromatograph equipped with a 5975C mass spectrometric detector was used for qualitative and quantitative analyses. Samples dissolved in diethyl ether:methanol (5:1, *v*/*v*) were applied (in a volume of 1–4 μL) using 1:10 split injection. The column used was a 30 m × 0.25 mm i.d., 0.25-μm, HP-5MS UI (Agilent Technologies, Santa Clara, CA, USA). Helium was used as the carrier gas at a flow rate of 1 mL/min. The separation was made either under isothermal conditions at 280 °C or in the temperature programmed: initial temperature of 160 °C held for 2 min, then increased to 280 °C at 5 °C/1 min and the final temperature of 280 °C held for further 44 min. The other employed parameters were as follows: inlet and FID (flame ionization detector) temperature 290 °C; MS transfer line temperature 275 °C; quadrupole temperature 150 °C; ion source temperature 230 °C; EI 70 eV; *m*/*z* range 33–500; FID gas (H2) flow 30 mL·min^−1^ (hydrogen generator); and air flow 400 mL·min^−1^. Individual compounds were identified by comparing their mass spectra with library data from Wiley 9th ED. and NIST 2008 Lib. SW Version 2010 or previously reported data and by comparison of their retention times and corresponding mass spectra with those of authentic standards, when available. Quantitation was performed using an external standard method based on calibration curves determined for the compounds belonging to representative triterpenoid classes: α-amyrin for triterpene alcohols, oleanolic acid methyl ester for triterpene acid methyl esters, and sitosterol for steroids.

### 4.11. Determination of Total Cadmium Concentration in Plant Tissues

To determine the total cadmium concentration in tissues, approximately 0.3 g of sample was digested in 10 mL of the oxidizing mixture: 9 mL of 69% HNO_3_ (Merck, Darmstadt, Germany) and 1 mL H_2_O_2_ (35%) at 180 °C for 30 min using the closed microwave system (Milestone Ethos Plus). Digested samples were transferred to plastic tubes and stored below 4 °C before measurement. The amount of cadmium was measured by Flame Atomic Absorption Spectroscopy (FAAS) using a Thermo Scientific—SOLAAR M Series (TJA Solution, SOLAAR M, UK) at the primary wavelength 228.8 nm. In FAAS the gas mixture was air and acetylene. The calibration curve range was 0–5 mg L^−1^ and the lower limit of quantification was 0.1 mg L^−1^. For background correction was used deuterium lamp (TJA Solution, SOLAAR M, UK). Cadmium standards solutions (Merck, Darmstadt, Germany) were prepared in 3% HNO_3_. The analysis was performed in Laboratory of Environmental Instrumental Analyzes at Faculty of Biology, University of Warsaw.

After determining the Cd concentrations in plants, the translocation factor (*TF*) was calculated using following equation:

$$TF=\frac{\mathrm{Cd}\;\mathrm{concentration}\;\mathrm{in}\;\mathrm{the}\;\mathrm{shoot}}{\mathrm{Cd}\;\mathrm{concentration}\;\mathrm{in}\;\mathrm{the}\;\mathrm{root}}$$

*TF* allows to determine heavy metal accumulation potential of plant. In plants showing features of hyperaccumulators the *TF* > 1 [18].

### 4.12. Statistical Analysis of Data

All data are presented as the means ± standard deviation of three independent samples analyzed in triplicate. For statistical analysis, t-Student’s test was applied using Microsoft Excel by Microsoft, Redmont, WA, USA, and STATISTICA by TIBCO Software Inc., Palo Alto, CA, USA. Statistical significance was considered at *p* < 0.05.

## 5. Conclusions

The report concerned the influence of cadmium stress on the steroid and triterpenoid metabolism in *Calendula officinalis* plants (roots and shoots) and hairy root cultures, and represented a targeted GC-MS metabolomic approach to study stress-induced metabolic modifications and shifts between general and specialized metabolism. The obtained results indicated that steroid and triterpenoid metabolism was significantly altered in *C. officinalis* plants and hairy root cultures exposed to Cd stress. The observed modifications involved the total content of these compounds, and particularly the proportions among individual sterols (e.g., stigmasterol-to-sitosterol ratio) and their ester and glycoside conjugates. The total sterol content increased in roots and hairy root culture, whereas it decreased in shoots; moreover, these effects were inversely correlated with Cd-induced growth suppression. The findings have suggested that various organs of the same plant can react differently to Cd stress, and sterols and their forms seemed to play a greater role in the response to Cd stress in roots than in shoots, whereas the enhanced synthesis of antioxidants like α-tocopherol was more visible in the aerial part of the plant. The symptoms of the competition between general metabolites (sterols) and specialized metabolites (triterpenoids) were also observed, i.e., the increase of the sterol biosynthesis parallel to the decrease of the triterpenoid content in *C. officinalis* plant roots and hairy root culture, and the inverse phenomenon, the decrease of the sterol content accompanied by the stimulation of triterpenoid biosynthesis in shoots.

The similarity of the metabolic modifications observed in the present study on *C. officinalis* plant roots and hairy root culture confirmed the possibility of application of plant in vitro cultures as convenient experimental models for physiological research on plant response to environmental stresses. However, the time-course and intensity of Cd stress response significantly differed in whole plants and hairy roots, therefore the investigation performed on in vitro culture should represent rather an initial approach for further studies in whole soil-growing plants.

## Data Availability

The data presented in this study are available in the article and Supplementary Materials.

## Supplementary Materials

The following supporting information can be downloaded at: https://www.mdpi.com/article/10.3390/ijms23105640/s1.
