# Supplementary material for: Modulation of Steroid and Triterpenoid Metabolism in Calendula officinalis Plants and Hairy Root Cultures Exposed to Cadmium Stress
Source: Int J Mol Sci. 2022 May 18;23(10):5640. doi: 10.3390/ijms23105640 (PMC9145312; doi:10.3390/ijms23105640)
Supplement: Supplementary file 1 [file ijms-23-05640-s001.zip › ijms-1729011-supplementary.pdf]

Modulation of steroid and triterpenoid metabolism in *Calendula officinalis* plants and hairy root cultures exposed to cadmium stress

Table S1. GC-MS data (retention times and characteristic ions of mass spectra) of identified steroids and triterpenoids.

Compound	Formula	Molecular weight	Retention time[min]	Mass spectrum *m/z*(relative intensity)
cholesterol	$C_{27}H_{46}O$	386.6	31.06	386 (26), 107 (50), 105 (48), 91 (57), 81 (54), 79 (46), 69 (47), 57 (87), 55 (73), 43 (100), 41 (55)
campesterol	$C_{28}H_{48}O$	400.6	33.59	400 (30), 107 (51), 105 (55), 95 (49), 83 (45), 81 (64), 71 (62), 57 (77), 55 (77), 43 (100), 41 (52)
stigmasterol	$C_{29}H_{48}O$	412.6	34.52	412 (36), 145 (64), 107 (52), 95 (100), 83 (66), 81 (90), 78 (60), 69 (67), 67 (85), 55 (69)
sitosterol	$C_{29}H_{50}O$	414.7	36.15	414 (29), 145 (54), 107 (59), 105 (60), 95 (54), 91 (49), 81 (57), 57 (68), 55 (70), 43 (100)
sitostanol	$C_{29}H_{52}O$	416.7	36.40	416 (31), 215 (82), 109 (58), 107 (83), 95 (81), 93 (64), 81 (84), 69 (60), 57 (64), 55 (81), 43 (100)
isofucosterol	$C_{29}H_{48}O$	412.3	36.78	412 (5), 314 (100), 105 (47), 95 (50), 91 (42), 83 (40), 81 (51), 69 (61), 55 (96), 43 (49)
β-amyrin	$C_{30}H_{50}O$	426.7	37.13	426 (27), 219 (18), 218 (100), 203 (49), 189 (17), 135 (11), 109 (13), 105 (12), 95 (15), 81 (18), 69 (14)
α-amyrin	$C_{30}H_{50}O$	426.7	38.62	426 (4), 219 (18), 218 (100), 203 (20), 189 (19), 135 (17), 133 (15), 122 (16), 119 (15), 95 (16)
tremulone	$C_{29}H_{46}O$	410.7	39.20	410 (32), 187 (27), 174 (100), 161 (37), 159 (26), 91 (28), 57 (28), 55 (37), 43 (44), 41 (28)
sitostenone	$C_{29}H_{48}O$	412.7	40.72	412 (37), 229 (34), 218 (31), 124 (100), 109 (31), 95 (41), 81 (27), 69 (32), 55 (37), 43 (44)
cycloartenol acetate	$C_{32}H_{52}O_2$	469.8	41.9	468 (24), 121 (20), 109 (32), 107 (29), 95 (41), 93 (24), 81 (27), 69 (30), 55 (23), 43 (100)
friedelinol	$C_{30}H_{52}O$	428.7	42.7	428 (3), 125 (45), 123 (49), 121 (48), 109 (75), 107 (47), 96 (68), 95 (100), 81 (66), 69 (82)
friedelin	$C_{30}H_{50}O$	426.7	43.7	426 (6), 125 (65), 123 (78), 109 (82), 107 (46), 96 (62), 95 (94), 81 (77), 69 (100), 67 (56)
oleanolic acid methyl ester	$C_{31}H_{50}O_3$	470.1	46.37	470 (1), 262 (48), 207 (13), 204 (16), 203 (100), 202 (21), 189 (22), 133 (17), 119 (13), 105 (14
stigmastane-3,6-dione	$C_{29}H_{48}O_2$	428.6	48.03	428 (25), 135 (61), 107 (74), 98 (63), 95 (67), 79 (62), 69 (86), 57 (67), 55 (100), 43 (77), 41 (71)
ursolic acid methyl ester	$C_{31}H_{50}O_3$	470.1	48.96	470 (1), 263 (20), 262 (100), 207 (32), 203 (93), 189 (29), 133 (76), 119 (34), 105 (21), 95 (18)

Table S2. Effect of cadmium treatment on steroid content in hairy roots tissue.

Compound	Content [µg/g DW± SD]							
	days							
	3		7		14		21	
	C	Cd	C	Cd	C	Cd	C	Cd
cholesterol	16.43±1.98 a	30.38±4.74 a	5.66±0.46 a	11.25±2.29 a	4.42±1.43 a	9.29±0.70 a	7.59±0.45 a	11.22±2.82 b
campesterol	39.74±2.91 a	82.10±8.95 b	42.61±4.15 a	57.84±4.91 a	80.01±9.16 a	67.60±0.34 a	72.72±2.30 a	62.07±5.36 a
stigmasterol	503.99±7.60 a	789.34±79.86 b	392.61±32.22 a	611.48±86.85 b	890.96±19.17 a	972.00±93.57 a	686.92±68.78 a	798.04±82.76 a
sitosterol	198.21±17.60 a	317.99±41.63 b	103.88±11.98 a	109.78±13.25 a	158.06±18.52 a	154.00±15.48 a	107.03±14.59 a	129.82±13.53 a
sitostanol	20.13±1.46 a	41.83±2.08 b	14.47±0.72 a	16.07±1.56 a	19.62±2.27 a	27.21±1.07 b	16.39±0.26 a	29.49±3.21 b
izofucosterol	28.61±3.91 a	65.13±5.42 b	16.70±2.25 a	28.70±2.33 b	53.89±6.85 a	57.86±4.27 a	42.11±5.92 a	39.04±2.15 a
tremulone	23.14±2.05 a	34.78±4.43 a	13.03±1.76 a	7.52±0.22 a	12.19±1.06 a	18.62±0.43 b	10.02±1.19 a	12.98±1.85 a
24-methylene cycloartenol	12.54±2.66 a	33.61±4.58 b	11.23±0.72 a	22.89±2.86 b	37.84±4.22 a	37.35±2.25 a	42.69±3.41 a	41.56±16.29 a
**Total**	**842.79**	**1395.15**	**600.20**	**865.52**	**1256.98**	**1343.94**	**985.48**	**1124.23**

Table S3. Effect of cadmium treatment on sterol esters in hairy roots tissue.

**Compound**	**Content [μg/g DW± SD]**							
	**days**							
	3		7		14		21	
	C	Cd	C	Cd	C	Cd	C	Cd
cholesterol	8.26±0.28 a	2.44±1.44 b	9.06±1.97 a	13.19±1.55a	10.75±2.64 a	23.56±4.13 b	18.26±3.52 a	23.18±0.06 a
campesterol	7.46±0.57a	2.61±1.93 b	7.40±1.94 a	9.92±2.51 a	18.43±5.69 a	11.15±5.04 a	19.08±2.63 a	25.09±1.35 b
stigmasterol	7.94±0.52 a	12.12±1.43 a	22.67±1.33 a	26.66±5.01 a	55.65±5.70 a	37.20±4.12 b	48.55±0.33 a	39.60±0.06 b
sitosterol	31.22±2.60 a	11.89±1.35 b	37.66±6.25 a	36.51±4.32 a	30.10±3.23 a	40.18±4.48 b	31.72±2.63 a	40.68±3.97 a
**Total**	**54.88**	**29.07**	**76.79**	**86.29**	**114.93**	**112.09**	**117.61**	**128.54**

Table S4. Effect of cadmium treatment on sterol glucosides in hairy roots tissue.

**Compound**	**Content [μg/g DW± SD]**							
	**days**							
	3		7		14		21	
	C	Cd	C	Cd	C	Cd	C	Cd
cholesterol	13.82±10.26 a	11.89±6.51 a	6.05±1.06 a	6.53±2.06 a	15.37±0.03 a	4.42±0.81 b	9.73±1.27 a	5.43±1.79 b
campesterol	80.2±7.65 a	77.94±16.19 a	49.18±1.81 a	40.34±10.47 a	40.5±6.65 a	22.35±0.74 b	48.16±5.91 a	49.22±6.68 a
stigmasterol	148.75±6.11 a	167.82±9.24 b	150.94±19.07 a	148.34±12.75 a	145.15±13.49 a	144.43±5.82 a	210.19±22.47 a	206.61±20.91 a
sitosterol	95.46±14.90 a	89.17±7.61 a	88.89±11.52 a	123.09±16.92 b	82.34±24.12 a	88.31±0.42 a	62.19±6.64 a	48.16±2.14 b
**Total**	**338.23**	**346.82**	**295.06**	**318.3**	**283.36**	**259.51**	**330.27**	**309.42**

TableS5. Effect of cadmium treatment on neutral terpenoid content in hairy roots tissue.

**Compound**	**Content [μg/g DW± SD]**							
	**days**							
	3		7		14		21	
	C	Cd	C	Cd	C	Cd	C	Cd
β-amyrin	32.80±2.96 a	64.48±5.31 b	33.63±4.64 a	30.43±1.97 a	14.72±2.12 a	18.20±0.36 a	26.53±4.57 a	19.98±2.87 a
α-amyrin	29.61±2.79 a	48.24±3.97 b	13.04±2.57 a	17.07±1.98 a	34.49±4.06 a	54.28±4.07 b	25.20±0.01 a	31.04±4.04 b
**Total:**	**62.41**	**112.72**	**46.66**	**47.51**	**49.21**	**72.48**	**51.74**	**51.02**

Table S6. Effect of cadmium treatment on free oleanolic acid (OA) content in hairy roots tissue.

Compound	Content [µg/g DW± SD]							
	days							
	3		7		14		21	
	C	Cd	C	Cd	C	Cd	C	Cd
OA	11.10±1.12 a	14.64±4.72 a	13.52±9.38 a	7.34±2.76 a	88.67±7.62 a	48.76±9.31 b	93.29±8.12 a	27.90±3.64 b

Table S7. Effect of cadmium treatment on oleanolic acid saponins (OA) content in hairy roots tissue.

Compound	Content [µg/g DW± SD]							
	days							
	3		7		14		21	
	C	Cd	C	Cd	C	Cd	C	Cd
OA	5022.95±255.43 a	5319.84±195.62 a	8182.88±1527.62 a	6084.22±998.08 a	8248.05±190.01 a	5284.42±803.04 b	10046.08±1337.51 a	3928.95±956.48 b

Table S8. Effect of cadmium treatment on oleanolic acid saponins (OA) released to the culture medium.

Compound	Content [mg/L•g DW± SD]							
	days							
	3		7		14		21	
	C	Cd	C	Cd	C	Cd	C	Cd
OA	0.37±0.10 a	0.20±0.13 a	0.30±0.00 a	0.19±0.08 a	0.38±0.24 a	3.07±0.39 b	4.10±0.99 a	2.03±0.47 b

Table S9. Content of free sterols, neutral triterpenoids and triterpenoid acids in *C. officinalis* roots.

Compound	Content [µg/g DW± SD]			
	Days			
	7		14	
	C	Cd	C	Cd
**Free sterols:**				
cholesterol	15.81±1.47 a	0	18.1±0.82 a	25.67±3.20 a
campesterol	141.42±13.53 a	142.41±1.31 a	71.04±8.62 a	84.58±9.29a
stigmasterol	924.76±29.42 a	907.82±81.50 a	445.91±47.83 a	371.93±40.81 a
sitosterol	491.78±48.02 a	618.58±32.31 b	255.91±28.83 a	304.43±31.22 a
sitostanol	138.87±13.39 a	81.62±9.66 b	95.52±8.46 a	239.26±2.25 b
tremulone	36.02±3.89 a	65.16±5.30 b	81.49±8.86 a	149.33±18.99 b
sitostenone	106.47±10.08 a	117.88±7.54 a	90.55±7.61 a	144.81±54.59 a
cycloartenol acetate	20.25±0.90 a	26.92±1.45 a	29.1±1.42 a	57.48±20.05 b
stigmastan-3,6-dione	0	0	26.25±2.30 a	73.32±10.55 b
**Total sterols:**	**1875.38**	**1960.39**	**1113.87**	**1450.81**
**Neutral triterpenoids:**				
β-amyrin	44.61±4.09 a	58.25±5.91 a	45.62±4.82 a	95.19±0.79 b
α-amyrin	37.48±1.27 a	42.51±4.93 a	40.85±5.85 a	69.18±4.90 b
**Sum of amyrins:**	**82.09**	**100.76**	**86.47**	**164.37**
fridelinol	67.29±6.96 a	83.44±2.19 b	76.21±1.43 a	112.38±0.27 b
friedelin	48.33±5.16 a	131.89±12.84 b	50.24±5.90 a	151.89±7.03 b
**Sum of friedooleanans:**	**115.6**	**215.33**	**126.45**	**264.27**
**Triterpenoid acids:**				
OA	32.54±2.50 a	36.12±3.41 b	33.26±1.14 a	30.50±4.16 b
UA	85.11±7.96 a	91.15±8.98 a	63.21±6.18 a	59.04±7.48 a
**Sum of triterpenoid acids:**	**117.65**	**127.27**	**96.47**	**89.54**

Table S10. Content of sterols conjugated in sterol esters and sterol glycosides in *C. officinalis* roots.

Compound	Content [µg/g DW± SD]			
	Days			
	7		14	
	C	Cd	C	Cd
**Sterol esters:**				
cholesterol	19.87±1.95 a	47.92±5.21 b	16.04±1.80 a	17.49±1.93 a
campesterol	58.62±6.25 a	67.26±10.34 a	22.01±3.22 a	66.04±7.13 b
stigmasterol	80.39±8.19 a	46.35±4.89 b	25.57±2.98 a	68.04±5.44 b
sitosterol	103.29±11.53 a	66.14±6.74 b	63.74±3.86 a	53.73±7.69 a
**Total:**	**262.17**	**227.67**	**127.36**	**205.3**
**Sterol glycosides:**				
cholesterol	43.67±7.62 a	53.65±0.86 b	22.99±0.29 a	27.02±1.47 b
campesterol	39.62±3.61 a	46.6±5.95 a	59.63±4.78 a	52.23±6.70 a
stigmasterol	55.88±4.26 a	63.08±3.23 a	50.46±5.46 a	52.17±7.10 a
sitosterol	105.38±0.22 a	136.62±10.34 b	77.69±3.43 a	85.71±3.79 b
**Total:**	**244.55**	**299.95**	**210.77**	**217.13**

Table S11. Contentof free sterols, neutral triterpenoids and triterpenoid acids in *C. officinalis* shoots.

**Compound**	**Content [µg/g DW± SD]**			
	**Days**			
	7		14	
	C	Cd	C	Cd
**Free sterols:**				
cholesterol	6.69±0.76 a	5.61±0.46 a	3.81±0.08 a	4.81±0.64 a
campesterol	32.84±4.84 a	22.49±0.55 a	24.02±3.18 a	18.55±2.71 a
stigmasterol	462.49±26.59 a	404.11±20.49 b	428.36±27.42 a	365.02±33.92 b
sitosterol	256.57±24.40 a	200.51±9.64 b	157.34±17.76 a	132.46±16.71 a
sitostanol	25.74±5.49 a	13.81±1.90 b	26.32±1.86 a	20.69±3.55 a
tremulone	7.04±0.67 a	12.89±1.04 b	7.79±0.86 a	8.45±1.25 a
sitostenone	17.04±0.05 a	20.55±2.95 b	9.05±0.13 a	11.46±1.25 a
**Total sterols:**	**808.41**	**679.97**	**656.69**	**561.44**
**Neutral triterpenoids:**				
β-amyrin	36.18±3.11 a	31.78±1.89 b	27.18±0.21 a	23.92±1.29 b
α-amyrin	49.61±4.85 a	47.31±2.35 a	39.85±2.35 a	43.41±3.20 a
**Sum of amyrins:**	**85.79**	**79.08**	**67.04**	**67.33**
**Triterpenoid acids:**				
OA	9.65±1.94 a	8.32±1.19 a	11.62±2.50 a	2.95±0.66 b
UA	24.28±2.96 a	15.88±1.24 b	15.58±0.13 a	6.04±0.51 b
**Sum of triterpenoid acids:**	**33.93**	**24.20**	**27.20**	**8.98**

Table S12. Content of sterols conjugated in sterol esters and sterol glycosides in *C. officinalis* shoots.

**Compound**	**Content [µg/g DW± SD]**			
	**Days**			
	7		14	
	C	Cd	C	Cd
**Sterol esters:**				
cholesterol	25.23±2.44 a	3.16±0.02 b	15.27±1.53 a	9.59±0.35 b
campesterol	42.55±6.92 a	29.01±2.37 b	26.55±2.34 a	16.47±1.93 b
stigmasterol	35.87±3.67 a	13.59±1.22 b	37.44±4.25 a	20.51±1.15 b
sitosterol	34.44±3.43 a	15.55±1.11 b	31.08±2.05 a	10.23±1.21 b
**Total:**	**138.09**	**61.31**	**110.34**	**56.8**
**Sterol glycosides:**				
cholesterol	38.25±2.86 a	43.17±4.17 a	21.42±0.29 a	26.62±2.02 b
campesterol	69.81±1.66 a	72.23±8.43 a	52.18±4.95 a	64.49±4.98 b
stigmasterol	128.05±17.97 a	203.53±6.49 b	144.17±13.38 a	137.25±12.57 a
sitosterol	95.37±9.21 a	118.06±10.43 b	134.64±14.09 a	130.03±15.67 a
**Total:**	**331.48**	**436.99**	**134.64**	**130.03**

Table S13. Content of oleanolic acid saponins (OA) in roots and shoots of *C. officinalis*.

**Organ**	**Content [µg/g DW± SD]**			
	**Days**			
	7		14	
	C	Cd	C	Cd
**Roots**				
OA	1200.03±131.54 a	2510.01±248.62 b	3133.85±476.44 a	5203.21±342.72 b
**Shoots**				
OA	14156.16±1356.23 a	16529.84±1156.34 a	18671.68±1978.37 a	20689.89±2456.59 a

Table S14. Basic physical and chemical charactrerization of Universal soil „Athena" including: pH, salinity, concentration of nitrogen (N), potassium oxide (K_2O) and phosphates (P_2O_5).

**Parameters**	**Units**	**„Athena" soil**
pH	-	5.5-6.5
Salinity	g/L	1.5
N	mg/L	180
K_2O	mg/L	220
P_2O_5	mg/L	160
